# 培美曲塞维持治疗恶性胸膜间皮瘤患者的疗效和安全性分析

**DOI:** 10.3779/j.issn.1009-3419.2021.101.48

**Published:** 2022-01-20

**Authors:** 晓梅 曾, 昭友 蒋, 建春 段

**Affiliations:** 1 610041 成都，成都市第七人民医院肿瘤科 Department of Oncology, Chengdu Seventh People's Hospital, Chengdu 610041, China; 2 100021 北京，中国医学科学院肿瘤医院内科 Department of Internal Medicine, Cancer Hospital Chinese Academy of Medical Sciences, Beijing 100021, China

**Keywords:** 恶性胸膜间皮瘤, 维持治疗, 培美曲塞, 无进展生存时间, Malignant pleural mesothelioma, Maintenance therapy, Pemetrexed, Progression free survival

## Abstract

**背景与目的:**

恶性胸膜间皮瘤（malignant pleural mesothelioma, MPM）是原发于胸膜且恶性程度高的一类肿瘤。晚期胸膜间皮瘤预后差，中位生存时间不超过15个月。一线标准化疗方案推荐是含培美曲塞的双药联合，加或不加贝伐珠单抗。目前一线标准化疗有效的患者能否从培美曲塞维持化疗中获益，尚无统一结论。本研究旨在探索培美曲塞单药维持在一线标准化疗有效的晚期MPM患者的疗效及安全性。

**方法:**

收集中国医学科学院肿瘤医院2013年1月-2018年1月确诊的一线培美曲塞联合铂类化疗4个-6个周期，且疾病未进展的MPM患者，共40例。根据患者后续是否进行培美曲塞单药维持治疗，将患者分为培美曲塞单药维持组（*n*=22）和观察组（*n*=18）。末次随访时间为2020年1月。主要研究终点为无进展生存时间（progression free survival, PFS），次要终点为总生存时间（overall survival, OS）、培美曲塞单药治疗的疗效以及不良反应发生情况。

**结果:**

培美曲塞维持治疗组较观察组中位PFS有所延长（8.5个月*vs* 3个月，*P*=0.008），而中位OS无明显差异（26.4个月*vs* 15.7个月，*P*=0.177）；两组的客观缓解率（objective response rate, ORR）分别为22.7%和0%。维持治疗组3级-4级不良反应包括1例（4.5%）患者出现4级中性粒细胞减少，1例（4.5%）患者出现3级中性粒细胞减少，1例（4.5%）患者出现4级贫血，3级恶心及食欲减低的发生率为4.5%。

**结论:**

培美曲塞单药维持治疗对于一线使用培美曲塞联合铂类化疗有效的MPM患者，中位PFS有获益，且耐受性可。

## Abstract (en)

**Background and objective:**

Malignant pleural mesothelioma (MPM) is a highly aggressive disease arising from pleural mesothelial cells. Advanced pleural mesothelioma has a poor prognosis, with a median survival of no more than 15 months. First line standard chemotherapy regimen recommended is Pemetrexed based chemotherapy regimen, with or without bevacizumab. There is no consensus on whether patients who have received first-line standard chemotherapy can benefit from pemetrexed maintenance chemotherapy. The study aimed to investigate the efficacy and safety of pemetrexed maintenance therapy (PMT) after treatment with a pemetrexed and platinum regimen for patients with MPM.

**Methods:**

A total of 40 MPM patients were collected from Cancer Hospital Chinese Academy of Medical Sciences from January 2013 to January 2018, eligible patients were unresectable MPM, without disease progression following 4 to 6 cycles of pemetrexed and platinum, including pemetrexed maintenance therapy group (22 cases) and observation group (18 cases). The last follow-up was conducted in January 2020. The primary endpoint were progression free survival (PFS), and the secondary end points were overall survival (OS), the efficacy, adverse reactions of PMT.

**Results:**

The median PFS in the PMT arm was longer than that in the observation arm (8.5 mon *vs* 3 mon, *P*=0.008), but there was no significant difference in median OS (26.4 mon *vs* 15.7 mon, *P*=0.177). Objective response rate (ORR) of two group were 22.7% and 0%, respectively. The grade 3-4 toxicity in PMT group included grade 4 neutropenia in 1 patient (4.5%), grade 3 neutropenia in 1 patient (4.5%), grade 4 anemia in 1 patient (4.5%) and grade 3 nausea and anorexia in 1 patient (4.5%).

**Conclusion:**

Pemetrexed maintenance therapy following initial pemetrexed and platinum chemotherapy improve PFS in patients with MPM, and is well tolerated.

恶性胸膜间皮瘤（malignant pleural mesothelioma, MPM）是原发于胸膜且恶性程度高的一类肿瘤，其发病率低，占所有恶性肿瘤的0.3%^[1]^。大多数患者确诊时已失去手术机会。晚期胸膜间皮瘤预后差，中位生存时间不超过15个月^[2]^。根据光镜下肿瘤细胞形态将其分为3种亚型：上皮样（占所有类型60%）、肉瘤样（占所有类型20%）及双相型（或混合型）（占所有类型20%）^[3]^。对于无法手术的患者，培美曲塞联合顺铂作为唯一的一线治疗方案长达20年之久^[4]^。在MAPS（Mesothelioma Avastin Cisplatin Pemetrexed Study）试验中，贝伐珠单抗加入标准一线治疗对比标准一线化疗，延长了患者中位生存时间（median overall survival, mOS）近3个月^[5]^。近年来，随着众多肿瘤从免疫检查点抑制剂（immune checkpoint inhibitors, ICIs）中获益，最新的研究显示程序性死亡配体1（programmed cell death ligand 1, PD-L1）抑制剂联合细胞毒性T淋巴细胞相关蛋白4（cytotoxic T lymphocyte-associated antigen 4, CTLA-4）抑制剂一线治疗晚期胸膜间皮瘤中上皮样亚型患者，相对标准化疗mOS有显著延长^[6]^。

对于一线治疗后肿瘤得到控制的患者，目前已证实维持治疗在非小细胞肺癌的治疗中取得了阳性结果。在MPM的治疗中，高效低毒化疗药物的维持治疗能否达到类似的疗效，尚无定论。因此，本研究旨在通过对近年来行培美曲塞维持治疗的MPM的患者进行回顾性分析，了解维持治疗在MPM患者的临床疗效及安全性，为临床治疗提供一定依据。

## 1 对象与方法

### 1.1 病例选择

我们收集了2013年1月1日-2018年1月1日于中国医学科学院肿瘤医院确诊的胸膜间皮瘤60例。纳入标准：①组织学或细胞学确诊为胸膜间皮瘤，不论手术或未手术，仍有靶病灶的患者；②一线治疗完成4个-6个周期培美曲塞联合铂类化疗；③一线化疗4个-6个周期后疗效评价为完全缓解（complete remission, CR）、部分缓解（partial response, PR）或疾病稳定（stable disease, SD）的患者；④末次化疗21 d-42 d内行培美曲塞单药维持治疗至少1个周期，维持治疗为每21 d-28 d为1个周期；或初始一线治疗结束后疾病进展前未行抗肿瘤治疗患者。排除标准：①合并其他恶性肿瘤或免疫系统、血液系统疾病；②各种原因引起的肝肾功能异常，存在肝肾功能严重障碍者。排除一线治疗后进展4例，诱导化疗周期数少于4个周期4例，以及资料不详尽者12例，最终纳入40例一线治疗有效患者进行回顾性分析，包括CR、PR及SD。

### 1.2 观察指标和疗效评定

本研究主要研究终点是经一线治疗后的无进展生存期（progression free survival, PFS），次要研究终点是维持治疗疗效、总生存时间（overall survival, OS），我们也观察收集维持化疗过程中药物不良反应的数据。PFS定义为从患者末次诱导化疗至患者出现疾病进展的时间。OS为患者末次诱导化疗至死亡的时间。诱导化疗期间每2个周期行胸腹部计算机断层扫描（computed tomography, CT）评估疗效。维持化疗过程中每2个-3个周期行胸腹部CT评估疗效。维持治疗组和观察组后续疗效评估的定义为维持治疗和观察期间最佳疗效与患者末次诱导治疗后的复查结果相比较。最佳疗效评价根据修改后实体瘤疗效评价标准（modified response evaluation criteria in solid tumors, mRECIST）标准。分期标准我们采用国际间皮瘤学会（International Mesothelioma Interest Group, IMIG）分期系统。不良反应采用不良事件常用术语标准（Common Terminology Criteria for Adverse Events, CTCAE）4.0版。

### 1.3 随访

所有患者均通过病历提供的联系方式进行电话随访，以行末次诱导治疗日期为随访起点，随访内容包括患者生存情况、疾病进展及治疗情况。按月计算，随访截止于患者死亡或者2020年1月1日。中位随访时间为20.1个月。失访患者、截至随访日期未发生疾病进展或者死亡的患者均定义为删失。

### 1.4 统计学方法

使用SPSS 22.0软件及R语言进行统计分析，两个或多个率之间的差异性分析采用*χ*^2^检验，生存和预后分析采用*Kaplan-Meier*法，差异性分析采用*Log-rank*检验；预后多因素分析用Cox比例风险模型，*P* < 0.05认为有统计学差异。

## 2 结果

### 2.1 患者基线特征

40例患者年龄范围在34岁-75岁，中位年龄为60.5岁；包括维持治疗22例，观察组18例。行手术治疗有6例，其中维持治疗组4例，观察组2例；3例行姑息放疗者均接受过培美曲塞维持治疗。两组患者基线特征无统计学差异。维持治疗组中维持化疗中位周期数是4个周期（1个-15个周期），其中1个-4个周期14例，6个-15个周期有8例，截至随访日期有3例患者仍在维持化疗中。21例患者一线治疗进展后行二线治疗，其中维持治疗组有8例，观察组13例。详见表 1。

**表 1:** 患者的临床特征 Clinical characteristics of patients

Characteristics	All patients (*n*=40)	Maintenance (*n*=22)	Observation (*n*=18)	*P*
Gender				0.919
Male	27 (67.5%)	15 (68.2%)	12 (66.7%)	
Female	13 (32.5%)	7 (31.8%)	6 (33.3%)	
Age (yr)				0.695
Median (range)	61.5（34-75）	61.5 (34-69)	61.5 (49-75)	
< 65	29 (72.5%)	17 (77.3%)	12 (66.7%)	
≥65	11 (27.5%)	5 (22.7%)	6 (33.3%)	
Smoking status				0.356
Smoker	19 (45.0%)	9 (40.9%)	10 (55.6%)	
Never smoker	21 (52.5%)	13 (59.1%)	8 (44.4%)	
IMIG stage				0.770a
Ⅰ	2 (5.0%)	1 (4.5%)	1(5.6%)	
Ⅱ	5 (12.5%)	2 (9.1%)	3 (16.7%)	
Ⅲ	9 (22.5%)	6 (27.3%)	3 (16.7%)	
Ⅳ	24 (60.0%)	13 (59.1%)	11 (61.1%)	
Histology				> 0.999b
Epithelial	35 (87.5%)	19 (86.4%)	16 (88.9%)	
Sarcomatoid	2 (5.0%)	1 (4.5%)	1 (5.6%)	
Mixed type	3 (7.5%)	2 (9.1%)	1 (5.6%)	
ECOG performance status				0.781
0	28 (70%）	15 (68.2%)	13 (72.2%)	
1	12 (30%）	7 (31.8%)	5 (27.8%)	
Best response to first-line standard chemotherapy				0.622
PR	15 (37.5%)	9 (40.9%)	6 (33.3%)	
SD	25 (62.5%)	13 (59.1%)	12 (66.7%)	
Prior surgery				0.859
No	34 (85.0%)	18 (81.8%)	16 (88.9%)	
Yes	6 (15.0%)	4 (18.2%)	2 (11.1%)	
Radiotherapy				0.305
No	37 (92.5%)	19 (95.5%)	18 (88.9%)	
Yes	3 (7.5%)	3 (13.6%)	0 (0.0%)	
IMIG: International Mesothelioma Interest Group; ECOG: Eastern Cooperative oncology Group; PR: partial response; SD: stable disease. a: *P* value was *χ*^2^ test of IMIG stage Ⅰ-Ⅱ *vs* Ⅲ-Ⅳ; b: *P* value was *χ*^2^ test of epithelial type *vs* non-epithelial type.				

### 2.2 疗效及预后

维持治疗组，一线治疗的客观缓解率（objective response rate, ORR）为40.9%，维持治疗期间ORR为22.7%，疾病控制率（disease control rate, DCR）为77.2%。观察组一线治疗ORR为33.3%，观察期间ORR为0%，DCR为55.6%，见表 2。维持治疗组的中位PFS明显长于观察组，分别为8.5个月（95%CI: 5.73-11.27）和3个月（95%CI: 0.01-7.11），有统计学差异（*P*=0.008）。两组的mOS相似：维持治疗组为26.4个月，观察组为15.7个月，无统计学差异（*P*=0.177），见图 1。

**表 2:** 患者的最佳疗效 Best response of patients

	All patients (*n*=40)	Maintenance (*n*=22)	Observation (*n*=18)
Best response to first-line standard chemotherapy			
PR	15 (37.5%)	9 (40.9%)	6 (33.3%)
SD	25 (62.5%)	13 (59.1%)	12 (66.7%)
Best response to maintenance group or observation group			
PR	8 (20.0%)	5 (22.7%)	0 (0.0%)
SD	19 (47.5%)	12 (54.5%)	10 (55.6%)
PD	13 (32.5%)	5 (22.7%)	8 (44.4%)
PD: progressive disease.			

**图 1:**
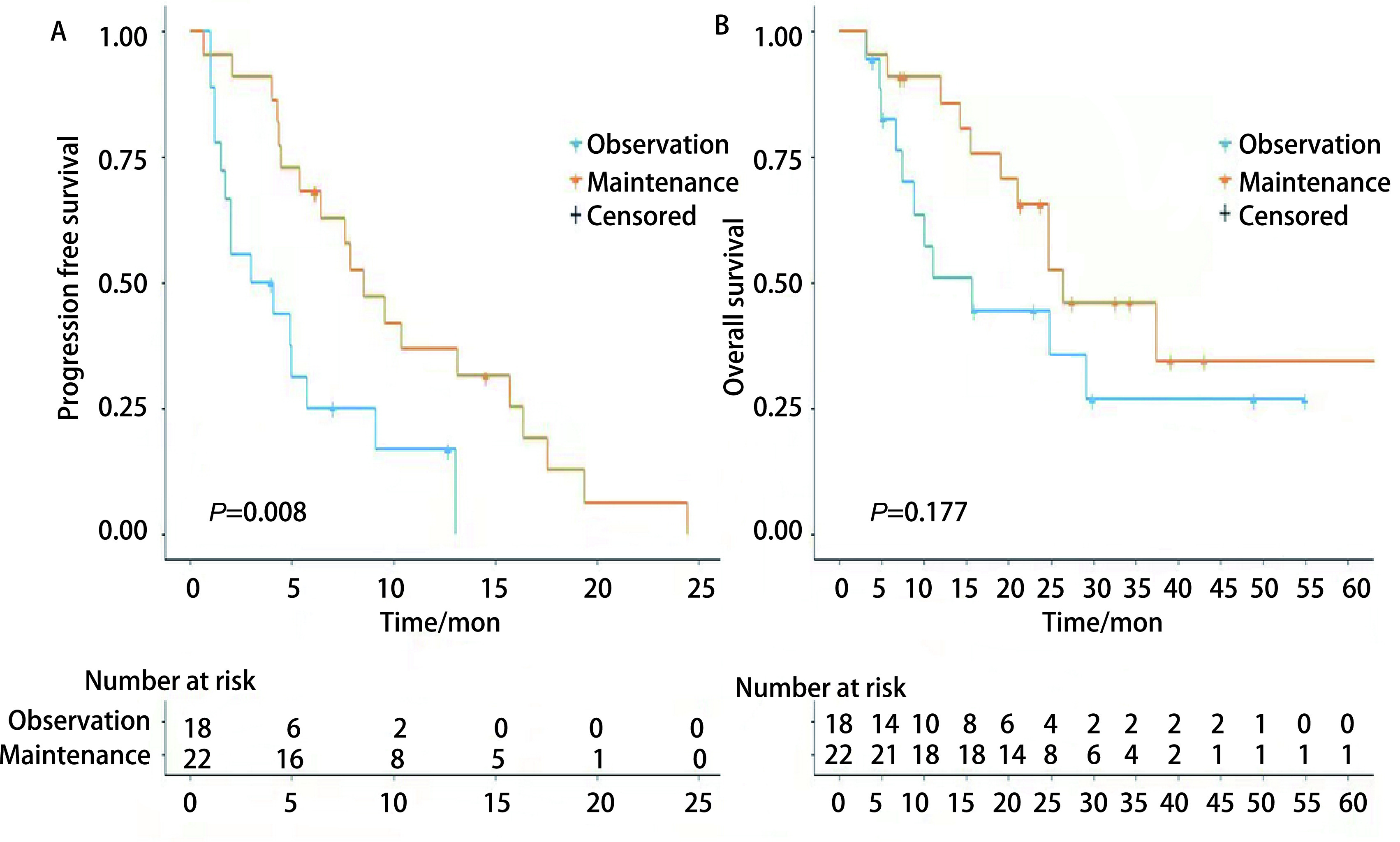
生存曲线。A：患者PFS的*Kaplan-Meier*曲线；B: 患者OS的*Kaplan-Meier*曲线。 Survival curve of the patients. A: Patient's progression free survival (PFS) curve; B: Patient's overall survival (OS) time curve.

将性别、年龄、美国东部肿瘤协作组（Eastern Cooperative Oncology Group, ECOG）体力状况评分、吸烟、病理类型（上皮型*vs*非上皮型）、分期（Ⅰ期-Ⅱ期*vs* Ⅲ期-Ⅳ期）、是否接受过手术治疗、是否接受过放疗等基线特征纳入单因素及多因素分析，单因素分析显示以上因素均未对PFS有影响，而单因素及多因素分析提示既往接受过手术的患者有更长的OS（*P*=0.015, *P*=0.034），多因素分析结果见表 3。

**表 3:** *Cox*生存分析患者的生存情况 *Cox* Proportional Hazards Modeling of patient's survival status

Influencing factors	PFS				OS		
	HR	95%CI	*P*		HR	95%CI	*P*
Gender	1.059	0.374-2.998	0.913		0.871	0.234-3.245	0.837
Age	1.054	0.449-5.039	0.509		1.232	0.319-4.761	0.762
ECOG performance status	0.689	0.300-1.579	0.378		0.558	0.196-1.590	0.275
Smoking status	0.967	0.306-3.055	0.955		1.709	0.328-8.913	0.525
Histology	0.313	0.092-1.063	0.063		2.471	0.486-12.564	0.276
IMIG stage	0.386	0.118-1.264	0.116		0.238	0.047-1.192	0.081
Prior surgery	2.686	0.837-8.615	0.097		9.456	1.185-75.449	0.034
Radiotherapy	0.252	0.056-1.141	0.074		0.564	0.092-3.467	0.537
Observation	5.049	1.768-14.422	0.002		2.558	0.826-7.919	0.103
PFS: progression free survival; OS: overall survival time; CI: confidence interval; HR: hazard ratio.							

### 2.3 不良反应

维持治疗期间，1例（4.5%）患者出现4级中性粒细胞减少，1例（4.5%）患者出现4级贫血，1例（4.5%）患者出现3级中性粒细胞减少，3级恶心或呕吐的发生率为4.5%。1级-2级贫血、中性粒细胞及白细胞减少的发生率分别为45.5%、45.5%及36.4%，1级肝功受损13.6%，1级-2级乏力40.9%，1级-2级消化道反应36.4%，皮疹及脱发的发生率均为4.5%，详见表 4。

**表 4:** 维持治疗组的不良反应 Adverse events in pemetrexed maintenance therapy group

		All	Grade 3	Grade 4
Hematologic adverse events				
Anemia		10 (45.5%)	0 (0.0%)	1 (4.5%)
Leucocytopenia		8 (36.4%)	0 (0.0%)	0 (0.0%)
Neutropenia		10 (45.5%)	1 (4.5%)	1 (4.5%)
Non hematologic adverse events				
Liver function injury		3 (13.6%)	0 (0.0%)	0 (0.0%)
Fatigue		9 (40.9%)	0 (0.0%)	0 (0.0%)
Nausea and vomit		8 (36.4%)	1 (4.5%)	0 (0.0%)
Constipation		1 (4.5%)	0 (0.0%)	0 (0.0%)
Rash		1 (4.5%)	0 (0.0%)	0 (0.0%)
Alopecia		1 (4.5%)	0 (0.0%)	0 (0.0%)

## 3 讨论

MPM是原发于胸膜间皮组织的一种少见肿瘤。有研究^[7]^发现，MPM发病与石棉暴露有关，男性发病率更高。MPM起病隐匿，大多数患者确诊时已是疾病晚期，其中位OS约1年，5年存活率约10%，治愈率极低^[8, 9]^。目前MPM的主要治疗方式包括手术、放疗、化疗、靶向治疗和免疫治疗。有研究^[10, 11]^发现，对于接受过手术、放疗及化疗三种治疗模式的患者，中位生存时间为20个月-29个月。在本研究中，有6例接受过手术联合化疗，但均为姑息性手术治疗，截至随访日期有3例存活，中位OS为35.9个月（23.8个月-63.2个月），本研究单因素和多因素分析均得出既往接受过手术治疗的患者有更长的OS，这可能与患者肿瘤负荷小才能接受手术，而接受手术治疗又大大减轻了患者的肿瘤负荷有关；有3例化疗联合姑息放疗，mOS为21个月（7.7个月-26.4个月）。Ⅰ期-Ⅱ期共7例患者，有1例Ⅱ期患者接受过手术治疗，4例患者合并胸腔积液及2例患者病灶较弥漫未行手术治疗，中位总生存时间（median overall survival, mOS）为24.8月；Ⅲ期-Ⅳ期患者，其中2例Ⅲ期（T3N0M0）和3例Ⅳ期（T4N0M0）患者接受过姑息性手术治疗，mOS为15.9个月，和以上文献数据基本相符。

培美曲塞联合顺铂作为MPM标准一线治疗方案，其有效率为41.3%^[4]^，本研究维持治疗组与未维持治疗组的诱导治疗的ORR分别是40.9%和33.3%，和前者基本相符。大多数患者经过一线治疗后出现疾病复发或进展，而二线治疗相关临床研究数据有限。虽然免疫检查点抑制剂纳武利尤单抗（Nivolumab）对比安慰剂治疗改善了复发的MPM预后^[12]^，但帕博利珠单抗（Pembrolizumab）对比化疗（吉西他滨或长春瑞滨）在MPM的二线治疗中未能显示出其优越性^[13]^。

对于一线标准化疗有效的MPM患者，维持化疗能否改善其预后，已有很多临床研进行了探索，包括原药（培美曲塞单药）维持以及换药维持治疗，目前仍无明确定论^[14-17]^。在Bogaert等^[14]^对27例患者进行的小规模试验中，在培美曲塞为基础的诱导治疗后，使用培美曲塞维持治疗患者较未接受维持治疗组，明显延长患者的疾病进展时间（time to progress, TTP）和OS（8.5个月*vs* 3.4个月和17.9个月*vs* 6个月，*P* < 0.000, 1），且毒性可耐受。我们研究中也观察到培美曲塞维持治疗增加了患者PFS的获益（8.5个月*vs* 3个月，*P*=0.008）；维持治疗组较观察组mOS有延长趋势（26.4个月*vs* 15.7个月，*P*=0.177），但无统计学差异。两组研究中，前者的TTP时间起点是从患者诱导治疗开始，而我们研究PFS定义为从诱导治疗开始到疾病进展或任何原因的死亡时间。结果显示本研究PFS及OS均长于前者。导致出现这种结果可能有以下原因：①两组研究的病例数均较少所致；②本研究中Ⅰ期和Ⅱ期患者占比较Bogaert等^[14]^的研究高（17.5% *vs* 11%），且部分患者接受过手术治疗，而此类患者预后较好，导致本研究中位PFS及OS较长。在最近一项Ⅱ期随机对照研究^[18]^中，纳入了49例不可切除的MPM，结果显示培美曲塞维持治疗组较观察组，患者的PFS和OS均无明显差异（3.4个月*vs* 3个月，*P*=0.973, 3；16.3个月*vs* 11.8个月，*P*=0.673, 7），结果与本研究不一致，可能与PFS及OS的定义不同，这项随机对照研究PFS为随机化到疾病进展或任何原因死亡的时间，OS为随机化开始至因任何原因引起死亡的时间，致PFS及OS较本研究短；其次入组的患者病例数有限，两组研究患者的基线情况有差异所致。这项试验中维持治疗组中3级-4级不良反应为白细胞减少（8%）、淋巴细胞减少（8%）、中性粒细胞减少（8%）以及疲乏（4%）。结果显示男性、放疗、ECOG评分差预示更短的PFS；OS的影响因素包括年龄、病理亚型、ECOG评分、有无胸痛及石棉暴露史。而我们研究中仅显示维持治疗延长患者PFS，既往接受过手术预示有更长的OS。在最新的NVALT19^[19]^中，证实了一线使用培美曲塞联合顺铂有效的MPM患者，更换为吉西他滨维持化疗对比观察组显著延长患者PFS（6.2个月*vs* 3.2个月，*P*=0.000, 2），3级-4级不良反应发生率分别为52%和16%。因此，一线标准化疗有效的MPM是否从维持化疗获益，还需大规模前瞻性随机临床试验来证实。

综上所述，培美曲塞单药维持治疗对于一线使用培美曲塞联合铂类化疗有效的MPM患者，有一定疗效，延长了患者的中位PFS，改善了患者的预后，且毒性低耐受较好。本研究进一步丰富了培美曲塞单药维持治疗在真实世界的临床研究数据，但目前尚缺乏相关大规模前瞻性临床试验。
